# *PORPHOBILINOGEN DEAMINASE* Deficiency Alters Vegetative and Reproductive Development and Causes Lesions in Arabidopsis

**DOI:** 10.1371/journal.pone.0053378

**Published:** 2013-01-08

**Authors:** Víctor Quesada, Raquel Sarmiento-Mañús, Rebeca González-Bayón, Andrea Hricová, María Rosa Ponce, José Luis Micol

**Affiliations:** Instituto de Bioingeniería, Universidad Miguel Hernández, Campus de Elche, Elche, Spain; Instituto de Biología Molecular y Celular de Plantas, Spain

## Abstract

The Arabidopsis *rugosa1* (*rug1*) mutant has irregularly shaped leaves and reduced growth. In the absence of pathogens, leaves of *rug1* plants have spontaneous lesions reminiscent of those seen in lesion-mimic mutants; *rug1* plants also express cytological and molecular markers associated with defence against pathogens. These *rug1* phenotypes are made stronger by dark/light transitions. The *rug1* mutant also has delayed flowering time, upregulation of the floral repressor *FLOWERING LOCUS C* (*FLC*) and downregulation of the flowering promoters *FT* and *SOC1/AGL20*. Vernalization suppresses the late flowering phenotype of *rug1* by repressing *FLC*. Microarray analysis revealed that 280 nuclear genes are differentially expressed between *rug1* and wild type; almost a quarter of these genes are involved in plant defence. In *rug1*, the auxin response is also affected and several auxin-responsive genes are downregulated. We identified the *RUG1* gene by map-based cloning and found that it encodes porphobilinogen deaminase (PBGD), also known as hydroxymethylbilane synthase, an enzyme of the tetrapyrrole biosynthesis pathway, which produces chlorophyll, heme, siroheme and phytochromobilin in plants. PBGD activity is reduced in *rug1* plants, which accumulate porphobilinogen. Our results indicate that Arabidopsis PBGD deficiency impairs the porphyrin pathway and triggers constitutive activation of plant defence mechanisms leading to leaf lesions and affecting vegetative and reproductive development.

## Introduction

Lesion-mimic mutants, which spontaneously develop necrotic leaf lesions similar to those caused by pathogen attack, have been identified in *Arabidopsis thaliana* and other plant species [1], [2]. The leaf damage in lesion-mimic mutants resembles the hypersensitive response (HR) that occurs during the plant response to an avirulent pathogen. The HR is triggered by resistance (R) proteins expressed by the host plant; these R proteins recognize specific avirulence (avr) factors expressed by the pathogen. As a consequence of avr recognition by R proteins, a signalling cascade is activated resulting in local cell death and rapid induction of plant resistance genes, finally leading to the activation of systemic acquired resistance (SAR), a broad-spectrum mechanism that confers resistance to further pathogen infection [3], [4]. Some lesion-mimic mutants constitutively express cytological and molecular markers associated with defence against pathogens and activated SAR [5].

**Figure 1:**
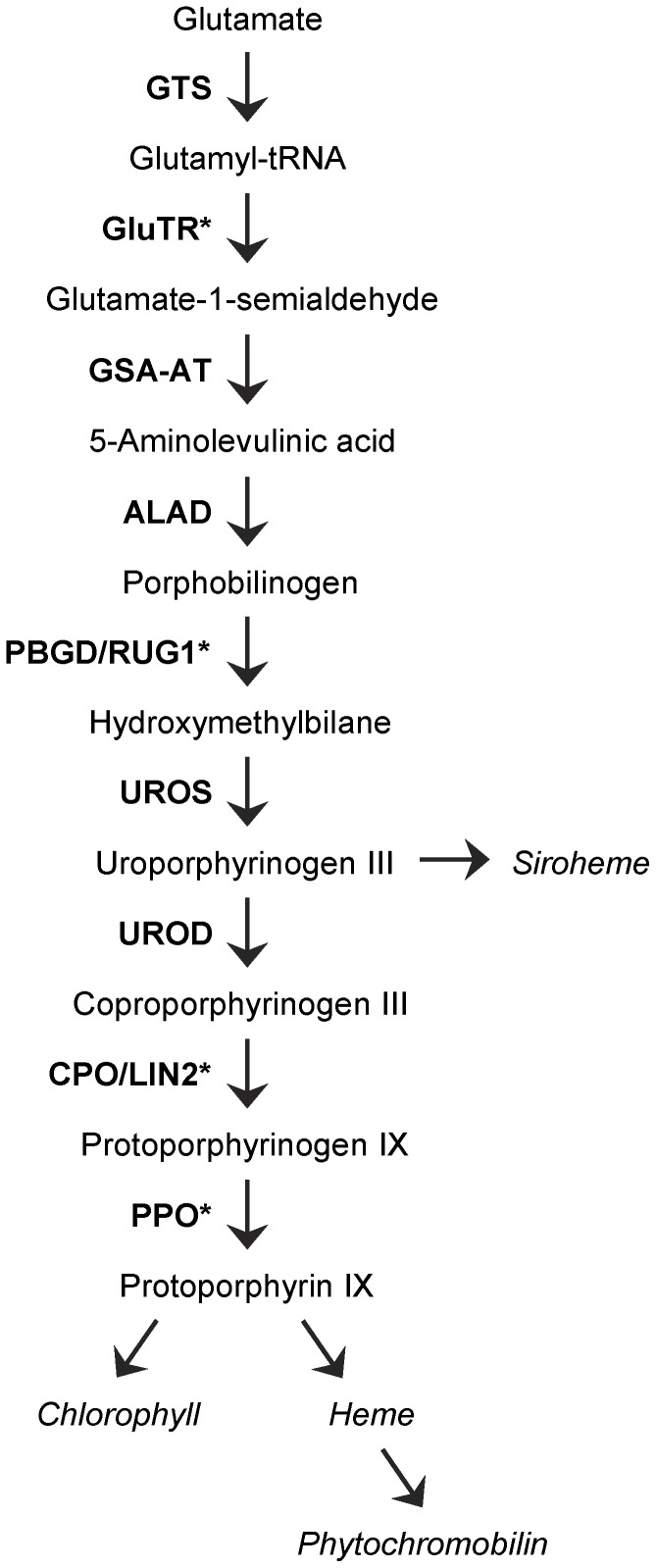
The tetrapyrrole biosynthetic pathway in plants. Enzymes (in bold capital letters) and intermediates of the nine steps of the common part of the pathway as well as the four end-products (in italics) are shown. GTS: Glutamyl-tRNA synthase; GluTR: Glutamyl-tRNA reductase; GSA-AT: Glutamate-1-semialdehyde amino-transferase; ALAD: 5-aminolaevulinic acid-dehydratase; PBGD: porphobilinogen deaminase; UROS: Uroporphyrinogen III synthase; UROD: Uroporphyrinogen III decarboxylase; CPO: coproporphyrinogen III oxidase; PPO: protoporphyrinogen III oxidase. Asterisks indicate genes for which a mutant phenotype has been reported in Arabidopsis. Redrawn from [23].

Several mutations causing lesion-mimic phenotypes have been cloned and some of these genes encode tetrapyrrole biosynthesis enzymes. For example, in maize necrotic leaf lesions are caused by loss of function of *Les22* (*Lesion mimic22*) and *cf1* (*camouflage1*), which encode urophorphyrinogen decarboxylase III (UROD) [6] and porphobilinogen deaminase (PBGD; also known as hydroxymethylbilane synthase; [7]), respectively. Also, Arabidopsis *LESION INITIATION2* (*LIN2*) encodes coproporphyrinogen III oxidase (CPO) [8] (Figure 1). Antisense-RNA mediated inhibition of genes encoding tetrapyrrole biosynthesis enzymes, such as the Arabidopsis glutamyl-tRNA reductase (GluTR; Figure 1) [9], and protoporphyrinogen IX oxidase (PPO; Figure 1) [10], can also cause lesion mimic phenotypes. Similarly, in *Nicotiana tabacum*, lesion mimic phenotypes are caused by RNA interference-mediated repression of CPO [11], [12], UROD [12], [13], PPO [14] and FeCh (ferrochelatase, an enzyme that acts in the heme branch of the tetrapyrrole biosynthesis pathway) [15].

The plastids of higher plants synthesize four classes of tetrapyrroles: chlorophyll, heme, phytochromobilin and siroheme, through a branched pathway whose enzymatic steps are well characterized [16], [17], [18] (Figure 1). Nearly 2% of Arabidopsis proteins bind tetrapyrroles, which act as cofactors in a number of fundamental biological processes such as photosynthesis, electron transport, oxygen transport and storage, detoxification, nitrogen fixation and light perception [17]. A role for tetrapyrrole biosynthesis intermediates, such as Mg-protoporphyrin IX, as retrograde signalling molecules transmitting information from the plastids to the nucleus to coordinate the expression of their genomes has also been proposed [19], but this hypothesis has been challenged [20], [21]. Recently, [22] reported that heme can act as a retrograde signalling molecule. These authors demonstrated that plastid ferrochelatase 1 (FC1, heme synthase) is overexpressed in the Arabidopsis *gun6* (*genomes uncoupled6*) mutant and that increased flux through the heme branch of the tetrapyrrole biosynthetic pathway enhances the expression of photosynthesis-associated nuclear genes. Some tetrapyrrole biosynthesis intermediates can produce reactive oxygen species when illuminated, mainly producing singlet oxygen; therefore, these intermediates are potentially toxic if they accumulate in excess [23]. Therefore, tetrapyrrole biosynthesis must be tightly regulated to adjust the production of its end products to the levels of cellular demand. For example, chlorophyll synthesis must be controlled in step with levels of chlorophyll apoproteins, to avoid chlorophyll excess and potential photodamage. The reduced amount of chlorophyll and the phototoxicity of tetrapyrrole intermediates in mutants defective in the porphyrin pathway could explain lesion formation in many lesion-mimic mutants.

Despite the above results, functional studies of mutants affected in some steps of the tetrapyrrole biosynthesis are still lacking. Here, we characterize the loss-of-function *rugosa1* (*rug1*) mutant of Arabidopsis; the leaves of *rug1* plants spontaneously develop small patches of necrotic tissue similar to those seen in lesion-mimic mutants. We cloned the *RUG1* gene and found that it encodes PBGD (Figure 1). Our results show that in Arabidopsis, disruption of the tetrapyrrole pathway at the step catalyzed by PBGD (polymerization of PBG to produce 1-hydroxymethylbilane) causes accumulation of PBG and directly or indirectly triggers the expression of plant defense genes, causes lesions and perturbs vegetative and reproductive development.

## Results

### The *rug1* Mutant Exhibits Necrotic Leaf Lesions

**Figure 2:**
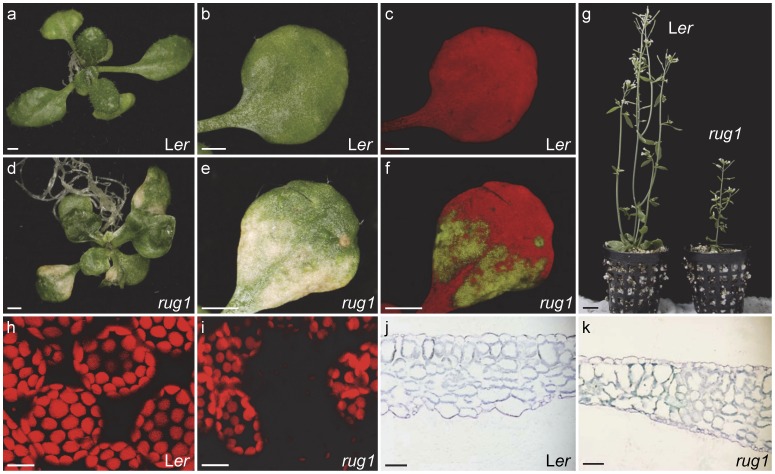
Lesion phenotype in the *rug1* mutant. (a, d) Three-week-old rosettes of the *rug1* mutant and the wild-type L*er*. (b, e) Close-up views of third-node vegetative leaves from the plants shown in panels (a) and (d). (c, f, h, i) Confocal micrographs showing fluorescing chlorophyll within mesophyll cells of (c, f) whole third-node leaves [those shown in (b) and (e)] and (h) details of the subepidermal layer of mesophyll cells of L*er* and (i) the boundary between a green and a pale sector in a *rug1* leaf. (g) 45-day-old plants grown in soil. (j, k) Transverse sections of third leaves. Bars = (a–f) 1 mm, (g) 1 cm, (h, i) 250 µm, and (j, k) 50 µm.

**Figure 3:**
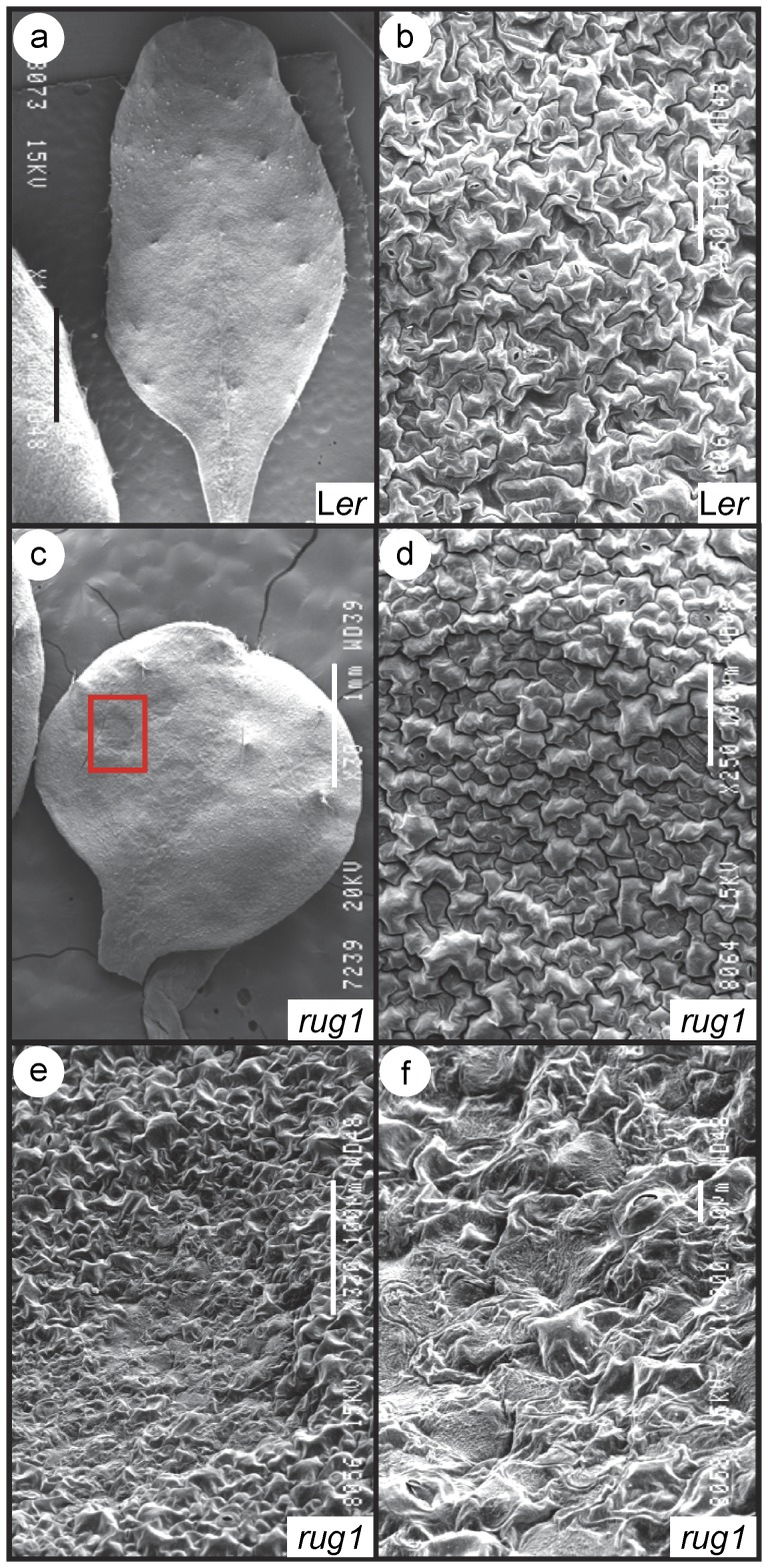
Scanning electron micrographs of *rug1* leaves. (a, c) Adaxial surface of third-node leaves and (b, d–f) details of the adaxial epidermis. (e, f) Different magnifications of the area boxed in red in (c), which corresponds to a necrotic sector. Pictures were taken 21 das (days after stratification). Bars = (a, c) 1 mm, (b, d, e) 100 µm, and (f) 10 µm.

The *rug1* mutant was isolated in a large-scale screen for EMS-induced Arabidopsis mutants with abnormal leaf morphology [24]. The recessive *rug1* mutation is expressed with complete penetrance and only minor variations in expressivity. The most eye-catching phenotype of *rug1* is the spontaneous development of lesions in its vegetative leaves; these lesions (Figure 2a, d) are visible to the naked eye as soon as 10 days after stratification (das). Lesions also occasionally occur in the cotyledons but not in other organs such as the main stem, cauline leaves, inflorescences or siliques. Lesion formation usually occurs as randomly distributed necrotic patches of leaf tissue, more numerous at the margin and apex, leading to pale and senescent areas that are visible on both the adaxial (Figure 2b, e) and abaxial surfaces. This phenotype resembles that previously described for Arabidopsis lesion-mimic mutants [25], which develop lesions in the absence of pathogens. This response resembles the HR elicited by inoculation with an avirulent pathogen or disease symptoms produced by pathogen attack. In addition to the lesion-mimic phenotype, *rug1* leaves are more irregular in shape than those of the wild-type Landsberg *erecta* (L*er*), display protruding leaf laminae and are usually curled up (Figure 2a, b, d, e). Scanning electron micrographs of the adaxial and abaxial epidermis of *rug1* leaves confirmed their irregularity (Figure 3a–d) and indicated that the lesion areas contained collapsed epidermal cells, a phenomenon not seen in other areas of the leaf (Figure 3e–f). Confocal microscopy and examination of transverse sections revealed that internal leaf structure was extremely perturbed in *rug1*: the lesion sectors lacked the chlorophyll autofluorescence normally exhibited by mesophyll cells (Figure 2c, f, h, i) and contained large air spaces (Figure 2h–k). No obvious alterations were found in other organs of *rug1*, although the mutant plants were of reduced height (Figure 2g).

### *rug1* is Similar to Lesion-mimic Mutants

**Figure 4:**
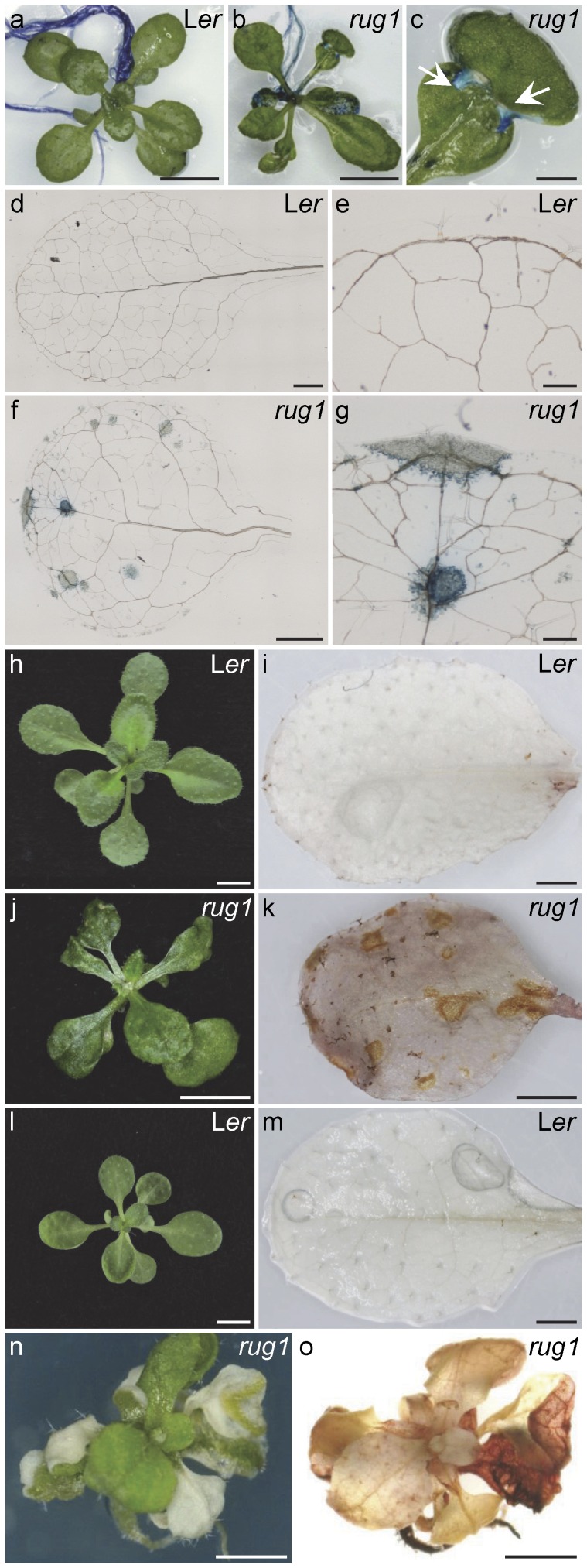
Lesion histology. (a–c) 21-day-old rosettes of (a) L*er* and (b) *rug1*, stained with toluidine blue, and (c) a third-node leaf from the plant shown in (b). Arrows in (c) indicate defective cuticle in a necrotic sector. (d–g) Trypan blue staining of (d) L*er* and (f) *rug1* third-node leaves and (e, g) close-up views of the leaves shown in (d) and (f), revealing dead cells in *rug1*. (h–o) (h, j, l, n) Rosettes of the genotypes indicated and (i, k, m, o) visualization of H_2_O_2_ accumulation by means of DAB staining of (i, k, m) one or (o) all of their leaves. Plants were grown under (a–k) continuous light or (l–o) long day conditions (16-h light/8-h dark). Bars = (a, b, h, j, l) 5 mm, (c, d, f, i, k, m–o) 1 mm, and (e, g) 200 µm.

The damaged areas of the leaves of lesion-mimic mutants express different cytological and molecular markers associated with the disease resistance response; plants undergoing HR after a pathogen attack also express these markers [5], [26], [27]. The similar lesion phenotypes of *rug1* and lesion-mimic mutants prompted us to investigate if some of these markers were expressed in the chlorotic areas of *rug1*. For this purpose, we stained *rug1* plants and leaves with toluidine blue (TB) to detect cuticle defects [28], diaminobenzidine (DAB) to detect H_2_O_2_ accumulation [29] and trypan blue (TP) to detect cell death [30]. TB staining revealed that areas of defective cuticle in *rug1* leaves overlap with chlorotic sectors (Figure 4a–c), and TP revealed areas of dead cells corresponding to lesions (Figure 4d–g). DAB treatment also detected H_2_O_2_ accumulation in the damaged areas of *rug1* leaves (Figure 4h–o); moreover, the sizes of the DAB-stained areas were much higher under 16-h light/8-h dark culture conditions (Figure 4l–o) than under continuous light (Figure 4h–k).

The accumulation of salicylic acid (SA) and the expression of some genes encoding pathogenesis-related proteins (PR) are associated with the formation of necrotic sectors in several lesion-mimic mutants and in wild-type plants infected by pathogens [31], [32]. To study whether these markers were also induced in *rug1*, we examined the expression of *PR1*, a classic marker for pathogen infection [5]. For that purpose, total RNA was extracted from 3-week-old L*er* and *rug1* plants, and we found by qRT-PCR that *PR1* was 5.7-fold upregulated in the mutant compared to L*er*. Accumulation of transcripts of *PR1* and of other genes involved in pathogen responses was also detected in our microarray analysis (see below). Given that SA induces *PR1* expression, we also measured by qRT-PCR expression of *SID2*, which encodes isochorismate synthase 1 (ICS1), the key enzyme in SA biosynthesis. We found that *SID2* was 1.5-fold overexpressed in *rug1* compared to L*er*. Taken together, our results show that *rug1* plants form lesions that phenocopy the effects of pathogen infection, as in other Arabidopsis lesion-mimic mutants.

### *rug1* is Late Flowering

We found that *rug1* plants flower moderately later than L*er* under continuous light (Figure S1a–c). The Arabidopsis MADS-box gene *FLOWERING LOCUS C* (*FLC*) is a potent repressor of flowering [33], [34]. Consistent with the delayed flowering phenotype, *FLC* was upregulated in the *rug1* mutants (Figure S1d). We also used qRT-PCR to measure the expression of the flowering-promoting genes *FLOWERING LOCUS T* (*FT*) and *SUPPRESSOR OF OVEREXPRESSION OF CONSTANS/AGAMOUS LIKE-20* (*SOC1/AGL20*), both of which are repressed by FLC. *FT* and *SOC1/AGL20* were downregulated in *rug1*, consistent with the late flowering phenotype and *FLC* overexpression detected in this mutant (Figure S1d).

Given that vernalization, the exposure to a long period of cold temperature (1 to 3 months at ∼1°C to 10°C), accelerates flowering in many Arabidopsis accessions and late flowering mutants [35], we also tested the vernalization response of *rug1* and found that the cold treatment induced L*er* and *rug1* plants to bolt earlier, suppressing the lateness of the *rug1* mutant (Figure S1b, c). Given that vernalization induces flowering by repressing *FLC* [35], we measured *FLC* expression in vernalized *rug1* plants and discovered a 10.8-fold reduction in *FLC* transcript levels compared to non-vernalized *rug1* plants.

### *RUG1* Encodes PBGD

**Figure 5:**
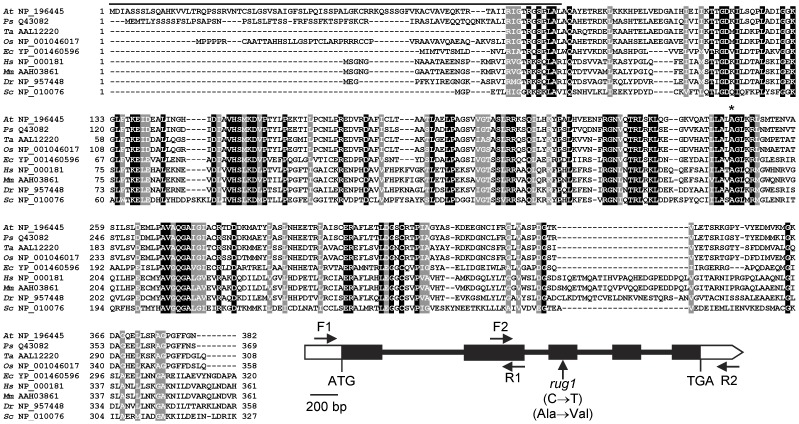
Conservation of PBGD and structure of the *RUG1* gene. Alignment of the predicted amino acids of the Arabidopsis RUG1 (NP_196445) protein with those of its putative orthologues from *Pisum sativum* (Q43082), *Triticum aestivum* (AAL12220), *Oryza sativa* (NP_001046017), *Escherichia coli* (YP_001460596), *Homo sapiens* (NP_000181), *Mus musculus* (AAH03861), *Danio rerio* (NP_957448) and *Saccharomyces cerevisiae* (NP_010076). Residues identical across all the sequences are shaded black; residues with similar chemical properties conserved across all five sequences are shaded grey. Numbers correspond to amino acid positions. Continuous lines indicate the N-terminal chloroplast transit peptide (as identified by the TargetP v1.0 program; [82]; http://www.cbs.dtu.dk/services/TargetP/). The alignment was obtained using ClustalX v 1.5b. The highly conserved amino acid that is changed in the *rug1* mutant is indicated by an asterisk. A schematic representation of the *RUG1* gene is also shown, with indication of the position of the *rug1* mutation. Exons and introns are represented by boxes and lines, respectively. White boxes correspond to the 5′ and 3′ untranslated regions. The predicted translation start (ATG) and stop (TGA) codons are indicated. Horizontal arrows, not drawn to scale, indicate the oligonucleotides used to characterize the structure of *RUG1*.

To better understand the function of *RUG1*, we used map-based cloning to identify the *RUG1* locus. The *RUG1* gene had been mapped at a low resolution [36]. Linkage analysis of an F_2_ mapping population derived from a cross of Col-0 to *rug1* (in the L*er* genetic background) allowed us to delimit a 54-kb candidate interval encompassing 19 annotated genes. We sequenced the transcription units of several genes within the interval and found a single difference between the *rug1* mutant and the wild-type L*er*: a C→T transition at position 1,212 (numbering from the predicted translation initiation codon; Figure 5) of the At5g08280 gene, which encodes porphobilinogen deaminase (PBGD; see below). The sequence change in *rug1* is predicted to cause an Ala→Val substitution in the RUG1 protein at position 246, a residue that is highly conserved among PBGDs (Figure 5). To confirm that the mutation in At5g08280 causes the phenotype of the *rug1* mutant, we complemented the mutant phenotype of *rug1* with a transgene carrying the *RUG1* wild-type coding sequence fused to the 35S promoter (Figure S2a–c; see Methods).

The *RUG1* open reading frame is predicted to encode a 382 amino acid protein of 41.04 kDa, porphobilinogen deaminase (PBGD; EC 2.5.1.61), which catalyzes the fifth enzymatic step of the tetrapyrrole biosynthesis pathway (Figure 1): the deamination and polymerization of four molecules of porphobilinogen in the linear tetrapyrrole 1-hydroxymethylbilane [37], [38]. PBGD has been purified from a wide-range of prokaryotic and eukaryotic organisms [39]. In animals and yeast, PBGD is a cytosolic protein but in higher plants and algae, it is targeted to the chloroplast [40]. In Arabidopsis, PBGD is a chloroplast protein encoded by a single-copy gene [41], [42]. The overall sequence similarity between the PBGD of Arabidopsis and other organisms is moderately high: 76, 75, 74, 46, 38, 37, 37 and 35% identity for pea, wheat, rice, *Escherichia coli*, human, mouse, *Danio rerio* and *Saccharomyces cerevisiae*, respectively (Figure 5). This is consistent with the properties of Arabidopsis PBGD, which is very similar to other PBGDs [39].

*RUG1* is broadly expressed, as shown by data deposited at different publicly available microarray databases [Genevestigator (https://www.genevestigator.com/gv/) and the BIO-array resource (BAR; http://bar.utoronto.ca/efp/cgi-bin/efpWeb.cgi)] and consistent with other experimental results that detected PBGD in different organs of Arabidopsis [41], [42] and pea [40]. Interestingly, we found that overexpression of Arabidopsis PBGD in a wild-type genetic background leads to the appearance of supernumerary shoot apical meristems and occasionally small necrotic patches in the leaves (Figure S2d–f).

### Light Affects the Phenotype of *rug1*

In maize, defective PBGD function causes the appearance of yellow sectors in the leaves of the *cf1* mutant under light/dark cycles, a phenotype that is suppressed when *cf1* plants are grown under continuous light [7]. Because we normally grow our plants under continuous light, we also tested whether growth under light/dark cycles could modify the *rug1* lesion phenotype. *rug1* plants grown under long-day conditions (16-h light and 8-h dark) displayed an apparent increase in the size of the chlorotic sectors and a reduction of plant growth compared to those grown under continuous light (Figure S3a, b, d, e). Remarkably, when *rug1* plants were grown under 16-h light/8-h dark conditions for 15 days followed by 8 days under continuous light, the lesion sectors were almost completely absent from the leaves (Figure S3c, f). These results indicate that sector formation in *rug1*, as in maize *cf1*, is dependent on the photoperiod conditions.

We also examined whether the lesion phenotype of *rug1* was affected by different light intensities by growing mutant and wild-type plants under light intensities lower (35 µmol m^−2^ s^−1^) and higher (115 µmol m^−2^ s^−1^) than those of our standard culture conditions (usually 65–70 µmol m^−2^ s^−1^). We found that the extent of the necrotic areas of *rug1* leaves were increased and reduced at the higher and lower light intensities, respectively (Figure S4a). We also grew *rug1* seedlings in the dark for 10 days to assess the photomorphogenic response of the mutant, but we observed no differences with L*er* (Figure S4b).

### PBGD and Catalase Activities are Reduced in *rug1*

**Figure 6:**
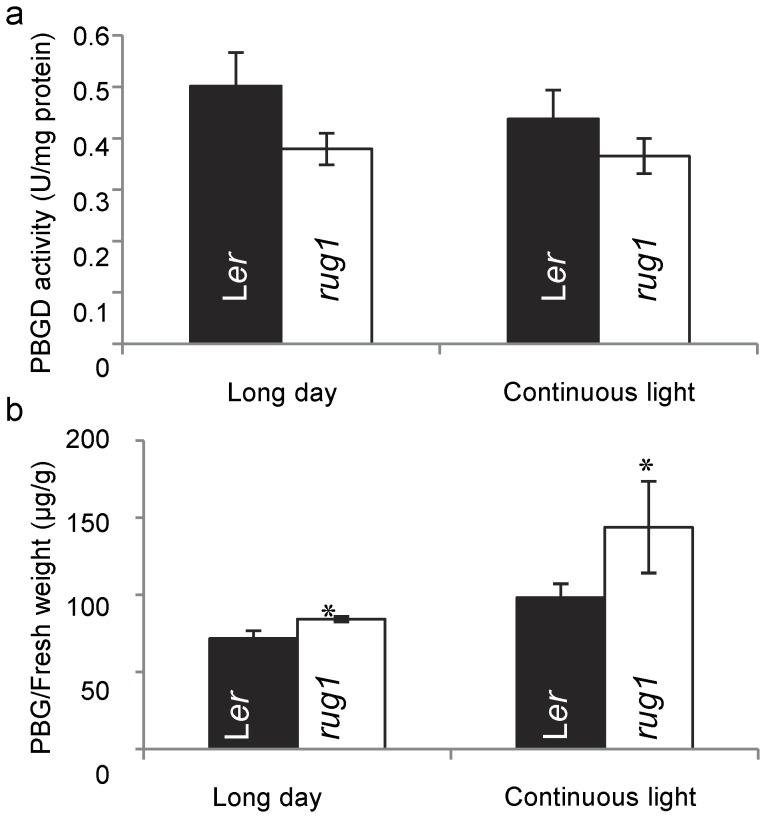
Measurements of PBGD activity and accumulation of PBG in *rug1* and L*er*. (a) PBGD activity in enzyme units per milligram of protein and (b) PBG accumulation in micrograms per gram of fresh weight in L*er* and *rug1* plants grown under long day conditions (16-h light/8-h dark) or continuous light. Asterisks indicate *rug1* values significantly different from those of the wild type (Students t-test, P<0.01).

To biochemically assess PBGD activity in *rug1*, extracts were obtained at 21 das from mutant and wild-type rosettes of plants grown under 16-h light/8-h dark photoperiod or continuous light conditions. Compared to L*er*, we detected a 24% reduction in PBGD activity in *rug1* under long day conditions and a 16% reduction under continuous light conditions (Figure 6a). Consistent with the decreased PBGD activity in *rug1*, the substrate of PBGD, porphobilinogen (PBG), accumulated in the mutant to levels significantly higher than in wild-type plants (Figure 6b). PBGD participates in the biosynthesis of heme, a cofactor of ROS scavenging enzymes such as catalase, and a defect in PBGD function in maize *cf1* causes a reduction in catalase activity [7]. To assess if the *rug1* mutation affects catalase, we measured this activity in mutant and wild-type rosettes. We found a moderate reduction in catalase activity in *rug1* plants grown under long day conditions (Figure S5).

### Auxin Response and Photoautotrophic Growth are Altered in *rug1*

Since several genes related to auxin signalling were downregulated in *rug1* (Table S1), we investigated whether the auxin response was altered in *rug1*. Root elongation was examined in *rug1* and L*er* plants grown on media supplemented with different indole-3-acetic acid (IAA) concentrations. The *rug1* plants had moderately reduced IAA sensitivity, revealing a relationship between porphyrin biosynthesis and auxin responsiveness (Figure S4c).

Given that PBGD participates in chlorophyll biosynthesis and that *rug1* exhibits a reduction in size, we also studied whether photoautotrophic growth was altered in the *rug1* mutants. To this end, *rug1* and L*er* plants were grown in culture media with or without 1% sucrose. We found that *rug1* growth was to some extent impaired when sucrose was not present: 12.5% of *rug1* seedlings were found to be developmentally arrested at the stage of green expanded cotyledons and first pair of tiny leaves versus 3.5% in L*er* (Figure S4d).

### Microarray and qRT-PCR Analyses of rug1

We also used microarray analysis to examine the effect of impaired *RUG1* function on expression of the Arabidopsis nuclear genome. We found 280 genes that were significantly misregulated, by at least 1.5-fold, in *rug1*, 173 (61.8%) upregulated and 107 (38.2%) down-regulated (Table S1). The genes were categorized either as known (233) or unknown (47) based on the annotations at the Arabidopsis Information Resource (TAIR; www.arabidopsis.org). The known genes were further classified into 13 different functional categories mainly based on the Functional Catalogue (FunCat) [43] assignments of the Munich Information Centre for Protein Sequencing (MIPS; http://mips.gsf.de) and literature reports [44] (Table S1). The largest categories identified were: “cell rescue, plant defence, senescence and virulence” (61 genes, 21.8%), “metabolism” (57 genes, 20.3%), “transcription” (28 genes, 10%) and “cellular communication/signal transduction” (23 genes, 8.2%). The main category includes genes encoding proteins involved in plant defence or resistance to pathogens, and most of these genes were overexpressed in *rug1* compared with L*er* (43 genes, 70.5%), consistent with the *rug1* lesion phenotype (Table S1). Thus, we identified proteins belonging to different plant pathogenesis-related (PR) families such as PR1, the plant defensin-fusion proteins PDF1.1, PDF1.2a, PDF1.2b, PDF1.2c, PDF1.3 and PDF1.4 (PR-12 family) [45], [46], [47], the lipid transfer protein 2 (LPT2; PR-14 family) [48], and a chitinase class IV protein (PR-3 family). This category also included the NPR1/NIM1 interacting protein NIMIN1 required for fine-tuning *PR1* expression [49], several members of the TIR-NBS family of plant disease resistance proteins (R proteins) [50] and the FLG22-INDUCED RECEPTOR-LIKE KINASE 1 (FRK1), whose expression is activated by bacterial flagellin and confers resistance to bacterial and fungal pathogens [51]. Other genes included in this category encoded proteins associated with senescence (SAG13 and SAG101) [52], [53], cell death [e.g. the ankyrin domain containing protein ACCELERATED CELL DEATH LIKE2 (ACL2) similar to ACD6, which activates SA-dependent cell death [54], detoxifying enzymes (P450 cytochromes, glutathione S-transferases, peroxidases and a heavy-metal-associated domain protein) or abiotic stress-responsive factors, such as the cold-responsive gene *KIN2/COR6.6* [55] and heat-shock factor 4.

In the “cellular communication” FunCat category, several putative signal transduction components were upregulated in *rug1*, including receptor-like protein kinases, protein kinases, calmodulin and calcium-binding proteins, which might potentially activate genes of the “cell rescue, plant defence, senescence and virulence” group. Within the “transcription” category, the most frequently represented transcription factor family was WRKY, whose members participate in pathogen defence, senescence, trichome development and biosynthesis of secondary metabolites [56]. The At2g46400 and At1g80840 genes, encoding WRKY46 and WRKY40 respectively, which are induced by the pathogen elicitor chitin [57] were up-regulated in *rug1*. The floral repressor *FLC* was the gene showing the largest fold-change in *rug1*, consistent with our qRT-PCR results and the late flowering phenotype of the mutant.

A total of 13 genes related to auxin response (included in the “systemic interaction with the environment” class) were misregulated in *rug1*, 12 of them belonging to the *SMALL AUXIN-UP RNA* (*SAUR*) family of auxin-inducible genes (Table S1), which are rapidly upregulated after auxin exposure [58]. The remaining gene, At5g13370, encoded a putative auxin-responsive GH3-like protein. Whereas all the *SAUR* genes were repressed in *rug1,* At5g13370 was upregulated.

We used the GOrilla web-based application (see Methods) for gene enrichment analysis in the *rug1* mutant. Significant enrichment was only shown when the “biological process” ontology was used but not with the “cell component” or “molecular function” options. The lowest P and false discovery rates (FDR) q values (2.58·10^−11^ and 6.04·10^−8^, respectively) among the down-regulated genes were attributed to “response to auxin stimulus” genes, all of them belonging to the SAUR family of auxin-inducible genes (see above; Figure S6 and Table S1). Other enriched processes were those of “root epidermal cell differentiation” (P = 5.04·10^−7^ and FDR q = 2.95·10^−4^) with 4 genes, and “anther development” (P = 1.6·10^−4^ and FDR q = 4.68·10^−2^) including 3 genes encoding glutaredoxins. Another functional enriched category was that of “response to stimulus”, including 24 genes, some of them belonging to the SAUR family; we did not took into account this group since large categories are typically not much informative. As regards the functional categorization of up-regulated genes, a more diverse scenario was found (Figure S6 and Table S1). The most significantly enriched processes that did not correspond to general (high-level) GO terms were those of response to fungi (P = 1.15·10^−10^ and FDR q = 4.47·10^−8^) and ethylene (P = 2.17·10^−10^ and FDR q = 7.25·10^−8^), with 9 genes included in each category, and innate immune response (P = 6.6·10^−10^ and FDR q = 1.71·10^−7^), with 12 genes. All of these categories shared some genes as with the salicylic acid related processes (Table S1).

To validate the results of our microarray experiment (Table S1), we chose some of the genes found misexpressed, to be analysed by qRT-PCR (Figure S1c). In *rug1* compared to L*er*, *FLC* and *PR1* were 19.0- and 5.7-fold up-regulated as measured by qRT-PCR, and 5.1- and 4.1-fold up-regulated, respectively, as measured by microarray (Table S1). In addition, qRT-PCR and microarray analyses showed 1.9- and 1.5-fold down-regulation, respectively, for *SOC1/AGL20*. Also, *PDF1.1* (At1g75830) and *SAG13* (At2g29350) were upregulated 10.4- and 7.4-fold by qRT-PCR, respectively, and 4.1-, and 3.9-fold by microarray analysis.

## Discussion

Nearly twenty years ago [41] isolated the Arabidopsis gene encoding PBGD. They found that it was a single copy gene in the Arabidopsis genome and that PBGD was targeted to chloroplasts. The same year, [39] published the purification and biochemical characterization of Arabidopsis PBGD. These authors discovered that Arabidopsis PBGD showed properties very similar to those of other prokaryotic and eukaryotic PBGDs, all of which were highly conserved. Despite the time elapsed, to date no work had been published on Arabidopsis PBGD function based on a mutational approach. Therefore, our study of the *rug1* mutant allows us, for the first time, to characterize at a genetic and molecular level the Arabidopsis gene encoding PBGD. Only one previous work described the cloning of a plant gene encoding PBGD from the isolation of a mutant: the maize non-clonal sectoring mutant *cf1* [7]. A likely explanation for the paucity of plant mutants affecting genes encoding PBGD is that they are single copy genes acting in a primary metabolic pathway, whose null alleles probably would be lethal. Hence, only hypomorphic alleles could be identified and studied.

In contrast with plants, a large amount of information is currently available about the effects of perturbed PBGD function in mammals, particularly in humans. Deficiency in PBGD produces acute intermittent porphyria (AIP), a severe and common form of the acute porphyrias, a group of inherited disorders caused by dysfunctions of the heme biosynthetic pathway in humans. AIP is associated with neuropathy attacks, including abdominal pain, vomiting and hypertension [59]. More than 300 mutations affecting human PBGD have been identified (The Human Gene Mutation Database; http://www.hgmd.cf.ac.uk/ac/gene.php?gene=HMBS), most of which are missense or nonsense mutations. A PBGD-defective mouse model has been developed that reproduces the neuropathic symptoms of human AIP [60]. Two major hypotheses have been invoked to explain porphyric neuropathy: (a) reduction in the levels of heme, and (b) direct toxicity caused by accumulated porphyrin precursors, including PBG.

*rug1* plants spontaneously develop chlorotic leaf lesions in the absence of pathogen attack, resembling the phenotype of lesion-mimic mutants. Like these, *rug1* exhibits cytological markers frequently associated with the formation of patches of dead tissues. Thus, the staining in *rug1* leaves of dead cells by TP and the detection of H_2_O_2_ by DAB in sites showing signs of damage before staining indicates that *rug1* plants form lesions similar to the HR caused by avirulent pathogens or disease symptoms following pathogen attack [25]. In Arabidopsis, lesion formation (named phytoporphyria in plants) [6] and the induction of defence responses caused by the inhibition of the activity of other enzymes of the tetrapyrrole pathway have been reported not only for PBGD but also for CPO [8] and PPO [10]. Interestingly, we discovered that overexpression of PBGD may lead to the formation of supernumerary apical meristems and the appearance of small patches of necrosis. This indicates that unbalanced porphyrin synthesis caused by either defective or enhanced activity of tetrapyrrole enzymes (such as PBGD) can have dramatic effects on plant development.

The phenotype of the Arabidopsis *rug1* mutant is similar to that of the maize *cf1* mutant. The similar phenotypic effect caused by defective PBGD in a monocotyledonous and a dicotyledonous species is consistent with the similarity between their amino acid sequences (71.6% identity and 90.6% similarity), biochemical activities and subcellular localization. Along these lines, PBGD activity was reduced in *cf1* and *rug1*, and both mutants exhibited increased porphobilinogen levels. Nevertheless, the reduction in PBGD activity was higher in *cf1* than in *rug1*, which is consistent with their molecular lesions, since *rug1* carries a missense mutation that affects a highly conserved residue of the RUG1 protein and *cf1* bears a *Mutator* transposon inserted in its 5′ UTR that strongly diminishes *CF1* expression [7].

Sectoring is notably enhanced in *rug1* plants grown under a light/dark cycle rather than under continuous light. [7] proposed a threshold model to explain the variegated phenotype of the *cf1* mutant of maize. According to this model, defective PBGD results in a reduction of the capacity to scavenge reactive oxygen species (ROS), especially in the bundle sheath cells, since heme is a cofactor of several ROS scavenging enzymes. As a consequence, an increase in cellular damage results in the formation of yellow sectors. The authors argue that lower levels of NADPH and antioxidant pools formed in the dark, together with the decreased ROS scavenging potential of *cf1* bundle sheath cells, would lead to a “burst” of oxidative damage upon illumination and thus trigger cell death. This would explain why yellow sectors form in dark/light cycles. Our experimental results showed that catalase activity is reduced in *rug1* plants grown under long day conditions (and hence exhibiting large chlorotic areas) as in *cf1* yellow sectors, supporting the model that reduced antioxidant activity is responsible for the formation of damaged areas. Nevertheless, contrary to *cf1* yellow sectors that do not accumulate H_2_O_2_ [61] *rug1* leaf lesions do accumulate H_2_O_2_. Hence, we cannot rule out the possibility that production of ROS caused by PBG accumulation might also contribute to lesion formation in *rug1*.

A photoperiod effect on the extent of the lesions in tetrapyrrole mutants has also been described for the Arabidopsis *lin2* mutant, which exhibits more severe lesions under long day than under short day conditions [8] and the *tigrina* (*tig*) mutant of barley, which accumulates the photosensitizer protochlorophyllide and shows sensitivity to dark/light cycles as do *rug1* and *cf1* [62].

Consistent with the lesion formation phenotype of *rug1* plants and constitutive activation of pathogenesis response mechanisms, our microarray analysis revealed that almost 300 genes were misregulated in the *rug1* mutant. The most abundant category was that of “cell rescue, plant defence, senescence and virulence”, and most genes in this category were over-expressed in the mutant. The SA-induced gene *PR1*, a marker of SA-dependent signaling [63], displayed the highest level of expression, as we confirmed by qRT-PCR experiments. *PR1* expression is a molecular marker of cytological damage and lesion-mimic mutants as well as wild-type plants infected by necrogenic pathogens [5], [27]. Besides, like *rug1*, Arabidopsis *lin2* plants affected in CPO accumulate *PR1* transcripts [8]. The increase in expression of *PR1* and *SID2*, which is involved in SA biosynthesis, point to an increase of SA activity in the *rug1* mutant. This would be expected since it is widely known that the concentration of SA, which is a signal required to elicit SAR, is high in lesion-mimic mutants such as *lin2* and increases after pathogen infection. Moreover, a role for SA in controlling flowering time in Arabidopsis has been proposed [64]. Along these lines, *rug1* plants flower later than the wild type, which is caused by the overexpression of the floral repressor FLC and the downregulation of the flowering promoting genes *FT* and *SOC1/AGL20*. This is in contrast with previous results showing that increased SA levels promote flowering in Arabidopsis by acting as a negative regulator of *FLC* expression [64]. A possible explanation for this discrepancy is that the end products or intermediates of the tetrapyrrole pathway might be required for SA to promote flowering in Arabidopsis. Accordingly, delayed flowering has also been reported for Arabidopsis antisense transgenic lines disrupted in the PPO tetrapyrrole enzyme, which exhibited high SA levels, accumulation of *PR1* transcripts and necrosis similar to that of *rug1* [10].

A connection between SA and auxin has been described in Arabidopsis and it has been proposed that pathogens can alter host auxin biosynthesis for their own benefit. In response, the host plants would be able to repress auxin signaling during infection by SA signaling [65], [66]. Thus, in a comprehensive study carried out to analyze the effects of SA on auxin signaling it was found that SA globally repressed auxin-related genes, thereby inhibiting auxin responses [66]. Interestingly, we found in our microarray analysis that 13 auxin-related genes were misregulated in *rug1*. Twelve genes encoding auxin-responsive proteins were repressed; some of these genes belong to the SAUR family, whose transcripts rapidly and transiently accumulate after auxin exposure [67]. The function of these genes, however, is largely unknown, likely due to genetic redundancy [68]. Interestingly, four of the *SAUR* genes were also repressed in wild-type Arabidopsis plants in response to an SA analog [66]. The remaining auxin-related gene was up-regulated and encoded a protein of the GH3 family, some members of which are IAA-amino acid conjugating enzymes [69]. Hence, auxin induction of genes of the GH3 family is assumed to diminish auxin signaling. Consistent with the hypothesis of auxin signaling being reduced in *rug1*, our root elongation assay indicated that *rug1* was more insensitive than the wild type to exogenous IAA.

In summary, a mutation in the PBGD gene of Arabidopsis has been reported for the first time. Our results reveal that, like in humans, perturbation of the tetrapyrrole pathway at the PBGD level severely disrupts cell metabolic homeostasis, leading to cell damage and even cell death, which has severe harmful effects on growth and development. The availability of the *rug1* mutant provides a valuable tool for further *in vivo* investigation on the function of plant PBGDs.

## Materials and Methods

### Plant Material, Growth Conditions and Growth Assays

Cultures and crosses were performed as described in [70] and [24], respectively. Seeds of the *Arabidopsis thaliana* (L.) Heynh. wild-type accessions Landsberg *erecta* (L*er*) and Columbia-0 (Col-0) were obtained from the Nottingham *Arabidopsis* Stock Centre (NASC). The *rug1* mutant was isolated in the L*er* background after ethyl methanesulfonate (EMS) mutagenesis and backcrossed twice to L*er* [24]. The *lin2* seeds were kindly provided by Atsushi Ishikawa. Light-sensitivity, autotrophic growth and photomorphogenic response analysis were performed as previously described [71]. Root growth inhibition by IAA was carried out as described in [72]. Plants were vernalized at the seed stage immediately after sowing on agar medium, for 4 weeks under continuous light at a temperature of 4°C±1°C. Flowering time was assayed by counting the total leaf number, rosette plus cauline, when the primary stem was above 5 cm tall as well as counting the number of days for bolting.

### Morphological, Histological and Biochemical Analyses

Whole rosette and single leaf pictures were taken in a Leica MZ6 stereomicroscope. For light microscopy, plant material was fixed with FAA/Triton (1.85% formaldehyde, 45% ethanol, 5% acetic acid and 1% Triton X-100) as described in [71]. 0.5-µm-thick transverse sections of leaves were cut on a microtome (Microm International HM350S), stained with 0.1% toluidine blue and observed using a Leica DMRB microscope equipped with a Nikon DXM1200 digital camera under bright-field illumination. Confocal imaging was performed as described in [73]. Trypan-blue (for cell death) and toulidine-blue (for cuticle defects) staining were performed as described in [30] and [28], respectively. Scanning electron microscopy was carried out as described in [71]. H_2_O_2_ was detected by DAB staining as described in [29]. PBG concentration and PBGD and catalase activity were measured from rosettes of the *rug1* mutant and the wild-type L*er* collected 21 das, as described in [7].

### Positional Cloning and Molecular Characterization of the *rug1* Mutations

To clone the *RUG1* gene, SSLP, SNP and CAPS markers were designed according to the polymorphisms between Landsberg *erecta* (L*er*) and Columbia (Col-0) described in the Monsanto *Arabidopsis* Polymorphism Collection database (http://www.arabidopsis.org). For allele sequencing, PCR products spanning the At5g08280 transcription unit were obtained using as a template wild-type and mutant genomic DNA and the oligonucleotide primers shown in Table S2 and Figure 5. Sequencing reactions were carried out with ABI PRISM BigDye Terminator Cycle Sequencing kits in 5-µl reaction volumes. Sequencing electrophoreses were performed on an ABI PRISM 3100 Genetic Analyzer.

### Complementation of the *rug1* Mutation and *RUG1* Overexpression

The coding region of At5g08280 was amplified by PCR using the attB-containing primers shown in Table S2 and a proofreading polymerase (Pfu Ultra; Stratagene). The product was firstly cloned into the pGEM-T Easy221 vector (kindly provided by B. Scheres) and then subcloned into the pMDC32 vector by recombination using Gateway technology (Invitrogen). Chemically competent *Escherichia coli* DH5α cells were heat-shocked and transformants were isolated and confirmed by PCR. Plasmid DNA was obtained and transformed into competent *Agrobacterium tumefaciens* LBA4404 cells. Positives clones containing the *35::RUG1* construct were used for *in planta* transformation of *rug1* and wild-type L*er* plants [74]. T_2_ seeds were sown in agar plates supplemented with 40 mg/ml of hygromycin for isolation of transformant plants. We used PCR to verify the presence of the transgene in the transformants.

### Quantitative RT-PCR

Total RNA was extracted from 50 to 70 mg of 3-week-old rosettes (L*er* and *rug1*) and DNase I treated using the Qiagen RNeasy Plant Mini Kit, following the manufacturer’s instructions. The RNA was reverse transcribed and subjected to qRT-PCR as described in [71]. Relative quantification of gene expression data was performed using the 2^−ΔΔC^_T_ or comparative C_T_ method [75]. Each reaction was performed in three replicates and levels of expression were normalized by using the C_T_ values obtained for the housekeeping gene *G3PDH*.

### Microarray Analysis

L*er* and *rug1* 3-week-old plants from 6 different sowings (80 to 100 mg per sample) were frozen in liquid N_2_ and ground by mortar and pestle. Total RNA was extracted as described in [76] and three biological replicates were obtained for each genotype by mixing two original RNA samples. 10 µg of total RNA from each biological replicate was used for microarray hybridization and analysis. In brief, Superamine Telechem slides containing more than 26,000 spots corresponding to the Arabidopsis oligo set from Qiagen-Operon, obtained from David Galbraith (Arizona University; http://ag.arizona.edu/microarray/), were hybridized by conventional methods with RNA probes labelled with either Cy3 or Cy5 Mono NHS Esters. For the hybridization, equal amounts of dye of each cDNA sample, ranging from 200 to 300 pmol, were mixed with the hybridization buffer containing 50% formamide, 3×SSC, 1% SDS, 5×Denhardt’s. This mixture was boiled for 5 minutes at 95°C and then added to the prehybridized slide. Hybridization took place overnight at 37°C in a hybridization chamber. Arrays were then washed in an orbital shaker for 5 min at 37°C in 0.5×SSC, 0.1% SDS; twice for 5 min at room temperature (RT) with 0.5×SSC, 0.1% SDS; three times with 0.5×SSC at RT, and 5 min with 0.1×SSC at RT. The slides were then spin-dried and scanned in a GenePix 4000B scanner (Axon Instruments) at 10 µm resolution, 100% laser power, and different PMT values to adjust the ratio to 1.0. Microarray images were analyzed using GenePix 5.1 (Axon Instruments) software.

The data were normalized and statistically analyzed using the LIMMA package [77], [78]. For local background correction the “normexp” method in LIMMA was used. The resulting log-ratios were print-tip loess normalized for each array. A multiple testing correction based on the false discovery rate (FDR) was performed to correct p-values. Genes were considered to be differentially expressed if the corrected p*-*values were <0.05 and their fold change greater than 1.5 fold or lower than −1.5 fold.

For gene enrichment analysis the GOrilla web-based application [79], [80] (http://cbl-gorilla.cs.technion.ac.il/) was used. Genes were classified into functional categories and visualized choosing two unranked (target and background) lists of genes as running mode. The background list was composed by all the genes on the array. P-values of 10^−3^ and 10^−5^ were selected as thresholds and the results obtained choosing three different ontologies (biological process, cell component and molecular function) were compared. The GOrilla tool transformed p-values into FDR q-values using the method described in [81].

## Supporting Information

Figure S1 **Flowering time in** ***rug1*** **.** (a) L*er* and *rug1* plants, pictured 33 das. Flowers and siliques are already visible in L*er* when bolting occurs in *rug1*. Bar = 1 cm. (b, c) Flowering time, determined as (b) the total leaf number (rosette and cauline leaves from the main inflorescence) and (c) the number of days for bolting. Both L*er* and *rug1* were grown under continuous light and vernalized for 4 weeks (Vernalization +) or just stratified (Vernalization −) before being transferred to our standard growth conditions. Values are means and standard errors for 20 plants. Asterisks indicate *rug1* values significantly different from those of L*er* (Students t-test, P<0.01). (d) qRT-PCR analysis of the expression of the *FLC, FT* and *SOC1* genes in the *rug1* mutant. Bars indicate relative levels of expression, determined as 2^−ΔΔC^_T_, for each of the studied genes after normalization with those of the housekeeping gene *G3PDH* and also normalized to the values obtained for L*er*, to which a value of 1 was given. All quantifications were made in triplicate on RNA samples. Plant material for qRT-PCR was collected 21 das
(PPT)

Figure S2 **Complementation of the mutant phenotype of** ***rug1*** **and effects of** ***RUG1*** **overexpression in a wild-type genetic background.** (a–e) Rosettes of (a) the *rug1* mutant, (b, c) transgenic plants carrying the *35S:RUG1* transgene in a *rug1* background, (b) one of which is phenotypically wild type while (c) the other does not show any of the mutant phenotypic traits that characterize *rug1* and develops many vegetative leaves, apparently as a consequence of shoot apical meristem duplication; (d, e) The phenotype shown in (c) was also caused by expression of the *35S:RUG1* transgene in a L*er* background (*RUG1*). (f) Some of these *35S:RUG1 RUG1* transgenic plants exhibited some necrotic spots. Pictures were taken (a, b) 21 das, (c) 29 das and (d–f) 26 das. Bars = 1 mm.
(PPT)

Figure S3 **Effect of different light conditions on the phenotype of the** ***rug1*** **mutant.** Rosettes of (a–c) L*er* and (d–f) *rug1* grown under (a, d) continuous light, (b, e) long day conditions (16-h light/8-h dark) and (c, f) 15 days in long day conditions followed by 8 days of continuous light. Pictures were taken at 23 das. Bars = 1 mm.
(PPT)

Figure S4 **Physiological analyses of the** ***rug1*** **mutant.** (a) Moderate light sensitivity of rug1 as seen by growing L*er* (upper panels) and *rug1* (bottom panels) under low (35 µmol m^−2^ s^−1^) or high (115 µmol m^−2^ s^−1^) levels of visible light. Arrows indicate enhanced necrotic lesions in *rug1* after exposure to high light intensities. (b) Skotomorphogenic growth is not altered in *rug1*. The histogram shows means (n≥15) and standard deviations of hypocotyl length in *rug1, lin2* and their respective wild types, L*er* and Col-0, grown in the dark for 10 days. Seedlings of the *aba1-1* and *aba1-101* mutants (in a L*er* and Col-0 genetic background, respectively) were included as controls since they are known to be partially defective in the skotomorphogenic response. The *lin2* mutant is deficient in the coproporphyrinogen III oxydase enzyme, which acts downstream of PBGD in the tetrapyrrole pathway (Figure 1). The *rug1* and *aba1-1* mutants are in the L*er* genetic background. *lin2* and *aba1-101* are in the Col-0 genetic background. (c) Root growth inhibition by IAA. Each point represents mean data (n≥15) of the reduction in root length displayed by plants grown on media supplemented with the IAA concentrations shown, compared with those grown on non-supplemented media. Error bars indicate standard deviations. Asterisks indicate *rug1* values significantly different from those of the wild type (Students t-test, P<0.01). (d) Effects of sucrose on *rug1* growth. L*er* (upper-left panel) and *rug1* (upper-right panel) plants grown in the absence of sucrose are shown. The bar graph represents the percentage of plants with arrested development in the absence of sucrose. Data are means of two different replicates of 50–100 seeds each, scored at 21 das. An arrested *rug1* seedling is marked by a red circle. Bars = (b) 1 mm and (d) 5 mm.
(PPT)

Figure S5 **Catalase activity in the** ***rug1*** **mutant.** Box plots showing catalase activity, expressed in enzyme units (U) per mg of protein. Samples were obtained from 21-day-old rosettes of the *rug1* mutant and its wild type L*er*, grown under continuous light or long day conditions (16-h light/8-h dark). Each box plot was obtained from the values of 3–6 measurements.
(PPT)

Figure S6 **GOrilla analysis output of** ***rug1*** **misregulated genes.** GO term enrichment for (a) down-regulated or (b) up-regulated genes using the Biological Process ontology is represented. Two unranked lists were used for enrichment calculations, consisting in genes represented in the microarray and recognized by the GOrilla database (18,726 in this study), and genes found down-regulated (103) or up-regulated (155) in the *rug1* mutant. Enrichment was calculated as (b/n)/(B/N). N: total number of genes in the reference set (microarray) associated with any GO term (16,222); B: number of genes in target set (64 and 73 down- and up-regulated genes, respectively, in the *rug1* microarray) associated with a GO Process; n: total number of genes in the microarray associated with a specific GO term, and b: number of (a) down- or (b) up-regulated genes in the *rug1* microarray associated with a specific GO term. Colors reflect the degree of GO term enrichment as indicated in the legend. A P-value of 10^−5^ was used as threshold.
(PPT)

Table S1 **Functional classification of misregulated genes in** ***rug1.***
(XLS)

Table S2 **Primers used in this work.**
(PDF)
