# Supplementary material for: PORPHOBILINOGEN DEAMINASE Deficiency Alters Vegetative and Reproductive Development and Causes Lesions in Arabidopsis
Source: PLoS One. 2013 Jan 8;8(1):e53378. doi: 10.1371/journal.pone.0053378 (PMC3540089; doi:10.1371/journal.pone.0053378)
Supplement: Figure S1 — Flowering time in rug1 . (a) Ler and rug1 plants, pictured 33 das. Flowers and siliques are already visible in Ler when bolting occurs in rug1. Bar = 1 cm. (b, c) Flowering time, determined as (b) the total leaf number (rosette and cauline leaves from the main inflorescence) and (c) the number of days for bolting. Both Ler and rug1 were grown under continuous light and vernalized for 4 weeks (Vernalization +) or just stratified (Vernalization −) before being transferred to our standard growth conditions. Values are means and standard errors for 20 plants. Asterisks indicate rug1 values significantly different from those of Ler (Students t-test, P<0.01). (d) qRT-PCR analysis of the expression of the FLC, FT and SOC1 genes in the rug1 mutant. Bars indicate relative levels of expression, determined as 2−ΔΔC T, for each of the studied genes after normalization with those of the housekeeping gene G3PDH and also normalized to the values obtained for Ler, to which a value of 1 was given. All quantifications were made in triplicate on RNA samples. Plant material for qRT-PCR was collected 21 das (PPT) [file pone.0053378.s001.ppt]

## Slide 1
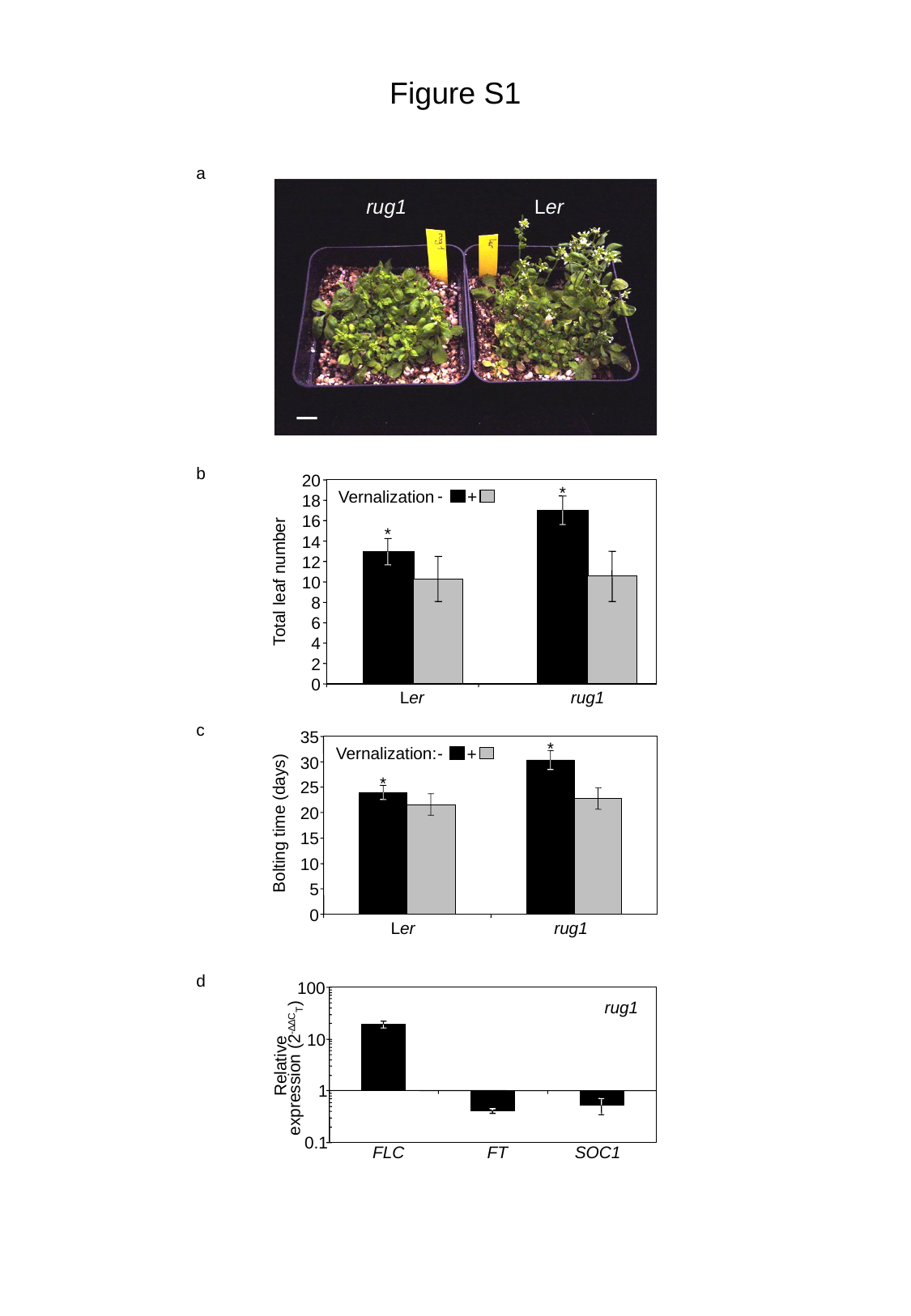

Figure S1
a
rug1
Ler
b
20
-
+
Vernalization
18
16
*
14
12
Total leaf number
10
8
6
4
2
0
Ler
rug1
c
35
-
+
Vernalization:
30
25
20
Bolting time (days)
15
10
5
0
Ler
rug1
d
100
rug1
10
Relative
expression (2-∆∆CT)
1
0.1
FLC
FT
SOC1
*
*
*
