# Supplementary figures and images for: PORPHOBILINOGEN DEAMINASE Deficiency Alters Vegetative and Reproductive Development and Causes Lesions in Arabidopsis
Source: PLoS One. 2013 Jan 8;8(1):e53378. doi: 10.1371/journal.pone.0053378 (PMC3540089; doi:10.1371/journal.pone.0053378)

## Slide 1
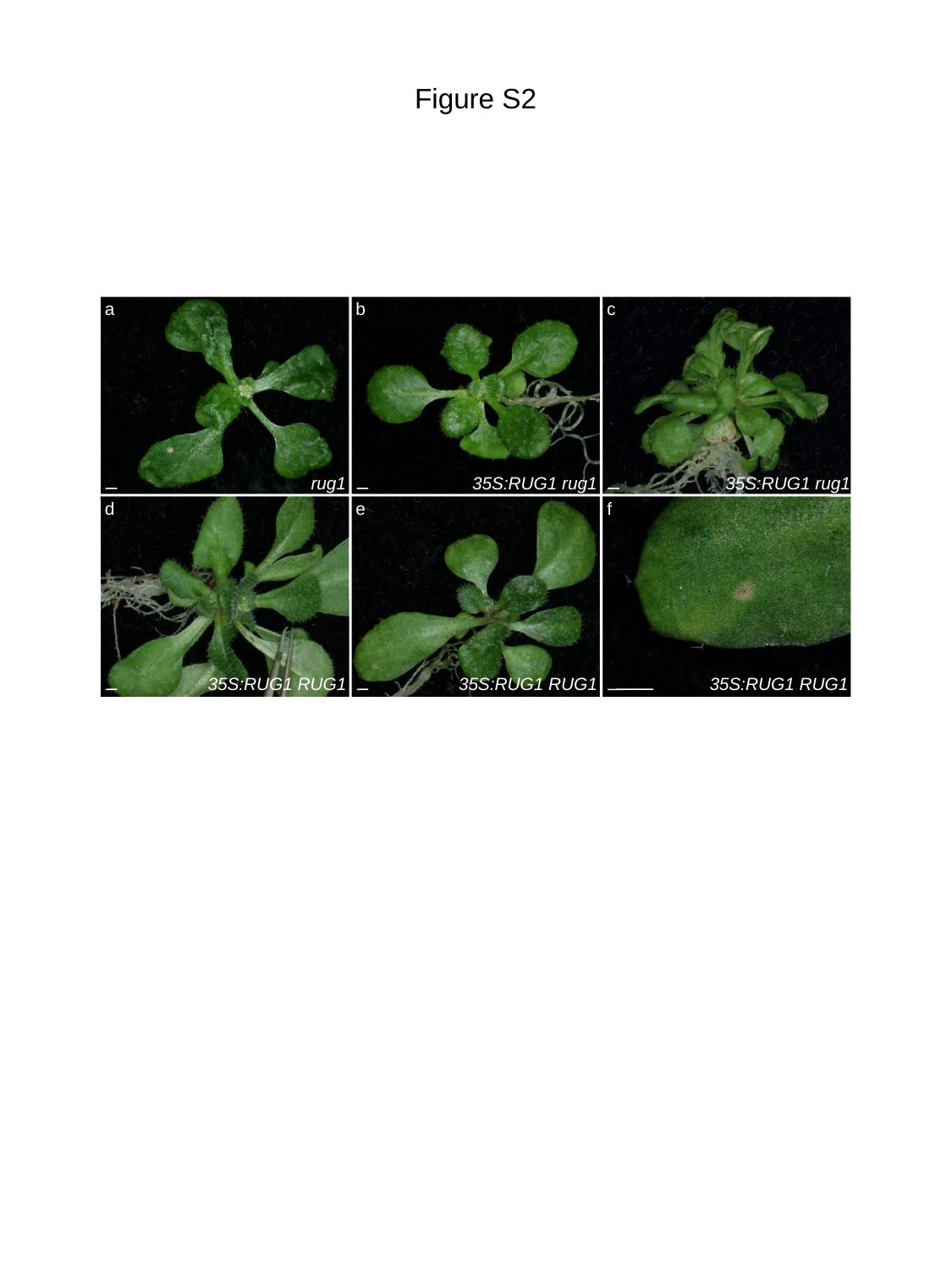

Figure S2
a
b
c
rug1
35S:RUG1 rug1
35S:RUG1 rug1
d
e
f
35S:RUG1 RUG1
35S:RUG1 RUG1
35S:RUG1 RUG1

Supplement: Figure S2 — Complementation of the mutant phenotype of rug1 and effects of RUG1 overexpression in a wild-type genetic background. (a–e) Rosettes of (a) the rug1 mutant, (b, c) transgenic plants carrying the 35S:RUG1 transgene in a rug1 background, (b) one of which is phenotypically wild type while (c) the other does not show any of the mutant phenotypic traits that characterize rug1 and develops many vegetative leaves, apparently as a consequence of shoot apical meristem duplication; (d, e) The phenotype shown in (c) was also caused by expression of the 35S:RUG1 transgene in a Ler background (RUG1). (f) Some of these 35S:RUG1 RUG1 transgenic plants exhibited some necrotic spots. Pictures were taken (a, b) 21 das, (c) 29 das and (d–f) 26 das. Bars = 1 mm. (PPT) [file pone.0053378.s002.ppt]

## Slide 1
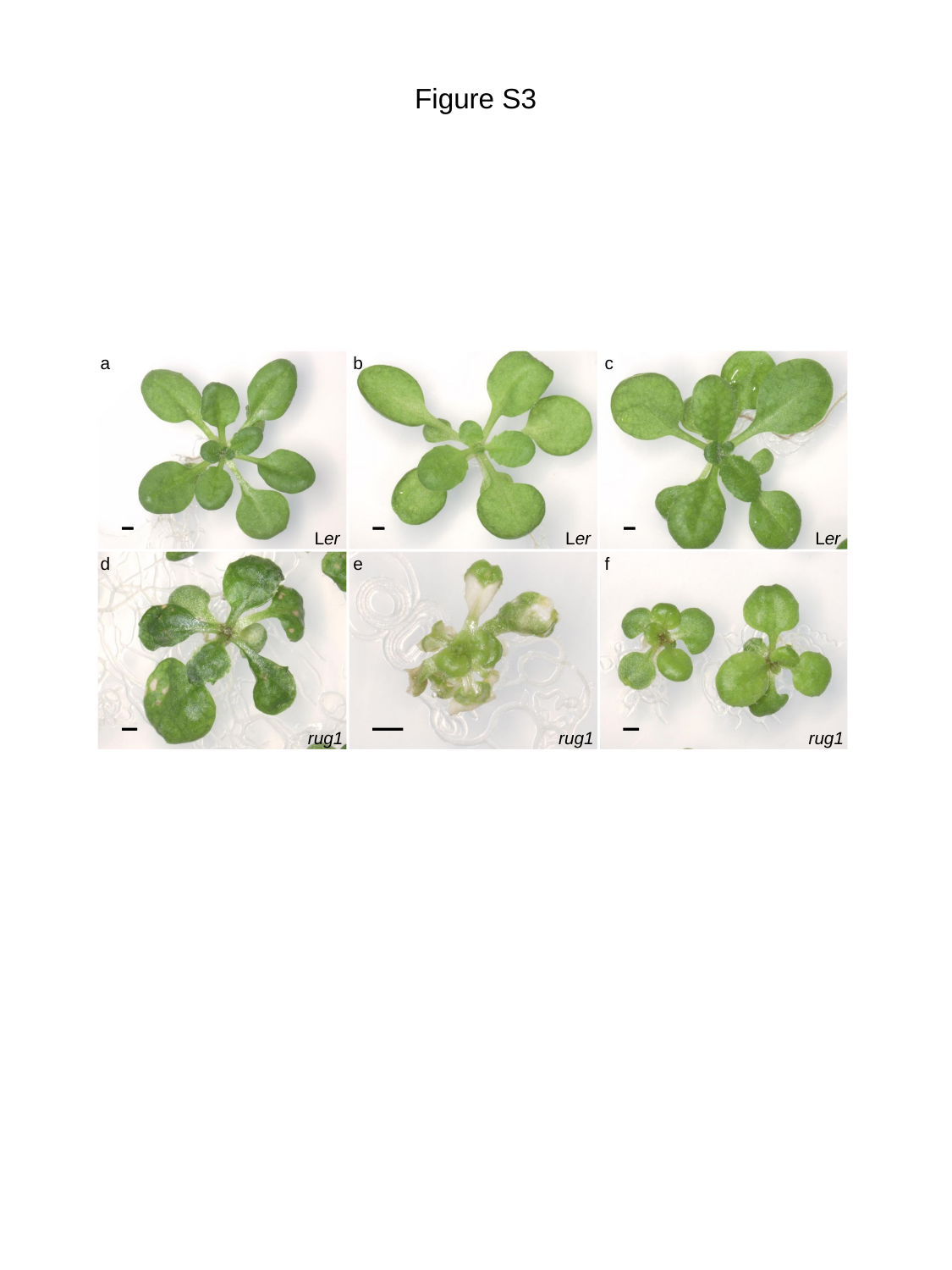

Figure S3
a
b
c
Ler
Ler
Ler
d
e
f
rug1
rug1
rug1

Supplement: Figure S3 — Effect of different light conditions on the phenotype of the rug1 mutant. Rosettes of (a–c) Ler and (d–f) rug1 grown under (a, d) continuous light, (b, e) long day conditions (16-h light/8-h dark) and (c, f) 15 days in long day conditions followed by 8 days of continuous light. Pictures were taken at 23 das. Bars = 1 mm. (PPT) [file pone.0053378.s003.ppt]

## Slide 1
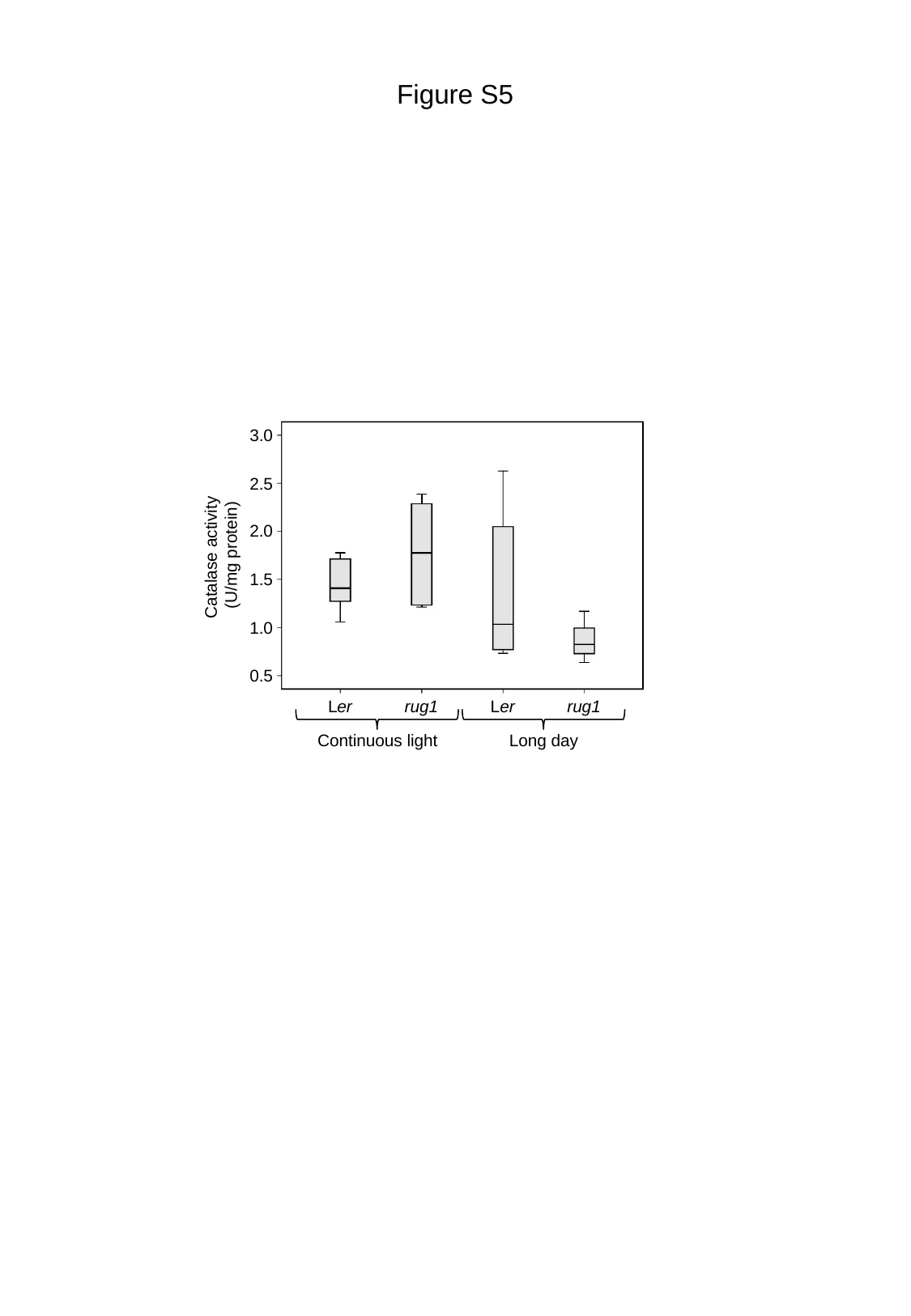

Figure S5
3.0
2.5
2.0
Catalase activity
(U/mg protein)
1.5
1.0
0.5
Ler
rug1
Ler
rug1
Continuous light
Long day

Supplement: Figure S5 — Catalase activity in the rug1 mutant. Box plots showing catalase activity, expressed in enzyme units (U) per mg of protein. Samples were obtained from 21-day-old rosettes of the rug1 mutant and its wild type Ler, grown under continuous light or long day conditions (16-h light/8-h dark). Each box plot was obtained from the values of 3–6 measurements. (PPT) [file pone.0053378.s005.ppt]
