# Supplementary material for: PORPHOBILINOGEN DEAMINASE Deficiency Alters Vegetative and Reproductive Development and Causes Lesions in Arabidopsis
Source: PLoS One. 2013 Jan 8;8(1):e53378. doi: 10.1371/journal.pone.0053378 (PMC3540089; doi:10.1371/journal.pone.0053378)
Supplement: Figure S4 — Physiological analyses of the rug1 mutant. (a) Moderate light sensitivity of rug1 as seen by growing Ler (upper panels) and rug1 (bottom panels) under low (35 µmol m−2 s−1) or high (115 µmol m−2 s−1) levels of visible light. Arrows indicate enhanced necrotic lesions in rug1 after exposure to high light intensities. (b) Skotomorphogenic growth is not altered in rug1. The histogram shows means (n≥15) and standard deviations of hypocotyl length in rug1, lin2 and their respective wild types, Ler and Col-0, grown in the dark for 10 days. Seedlings of the aba1-1 and aba1-101 mutants (in a Ler and Col-0 genetic background, respectively) were included as controls since they are known to be partially defective in the skotomorphogenic response. The lin2 mutant is deficient in the coproporphyrinogen III oxydase enzyme, which acts downstream of PBGD in the tetrapyrrole pathway (Figure 1). The rug1 and aba1-1 mutants are in the Ler genetic background. lin2 and aba1-101 are in the Col-0 genetic background. (c) Root growth inhibition by IAA. Each point represents mean data (n≥15) of the reduction in root length displayed by plants grown on media supplemented with the IAA concentrations shown, compared with those grown on non-supplemented media. Error bars indicate standard deviations. Asterisks indicate rug1 values significantly different from those of the wild type (Students t-test, P<0.01). (d) Effects of sucrose on rug1 growth. Ler (upper-left panel) and rug1 (upper-right panel) plants grown in the absence of sucrose are shown. The bar graph represents the percentage of plants with arrested development in the absence of sucrose. Data are means of two different replicates of 50–100 seeds each, scored at 21 das. An arrested rug1 seedling is marked by a red circle. Bars = (b) 1 mm and (d) 5 mm. (PPT) [file pone.0053378.s004.ppt]

## Slide 1
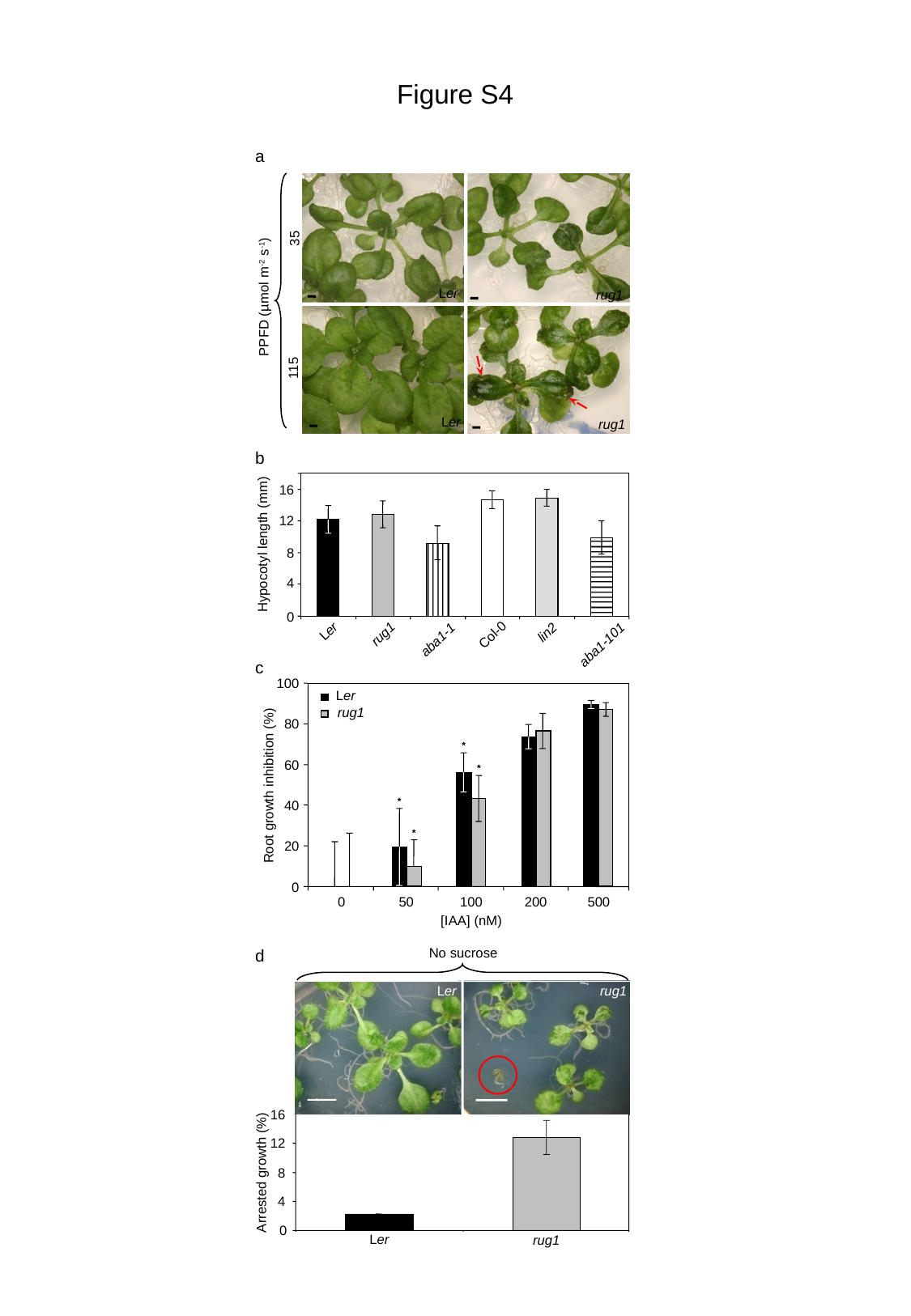

Figure S4
a
35
Ler
rug1
 PPFD (µmol m-2 s-1)
115
Ler
rug1
b
16
12
Hypocotyl length (mm)
8
4
0
Ler
lin2
rug1
Col-0
aba1-1
aba1-101
c
100
Ler
rug1
80
*
*
60
*
Root growth inhibition (%)
40
*
20
0
0
50
100
200
500
[IAA] (nM)
No sucrose
d
Ler
rug1
16
12
Arrested growth (%)
8
4
0
Ler
rug1
