# Supplementary material for: PORPHOBILINOGEN DEAMINASE Deficiency Alters Vegetative and Reproductive Development and Causes Lesions in Arabidopsis
Source: PLoS One. 2013 Jan 8;8(1):e53378. doi: 10.1371/journal.pone.0053378 (PMC3540089; doi:10.1371/journal.pone.0053378)
Supplement: Figure S6 — GOrilla analysis output of rug1 misregulated genes. GO term enrichment for (a) down-regulated or (b) up-regulated genes using the Biological Process ontology is represented. Two unranked lists were used for enrichment calculations, consisting in genes represented in the microarray and recognized by the GOrilla database (18,726 in this study), and genes found down-regulated (103) or up-regulated (155) in the rug1 mutant. Enrichment was calculated as (b/n)/(B/N). N: total number of genes in the reference set (microarray) associated with any GO term (16,222); B: number of genes in target set (64 and 73 down- and up-regulated genes, respectively, in the rug1 microarray) associated with a GO Process; n: total number of genes in the microarray associated with a specific GO term, and b: number of (a) down- or (b) up-regulated genes in the rug1 microarray associated with a specific GO term. Colors reflect the degree of GO term enrichment as indicated in the legend. A P-value of 10−5 was used as threshold. (PPT) [file pone.0053378.s006.ppt]

## Slide 1
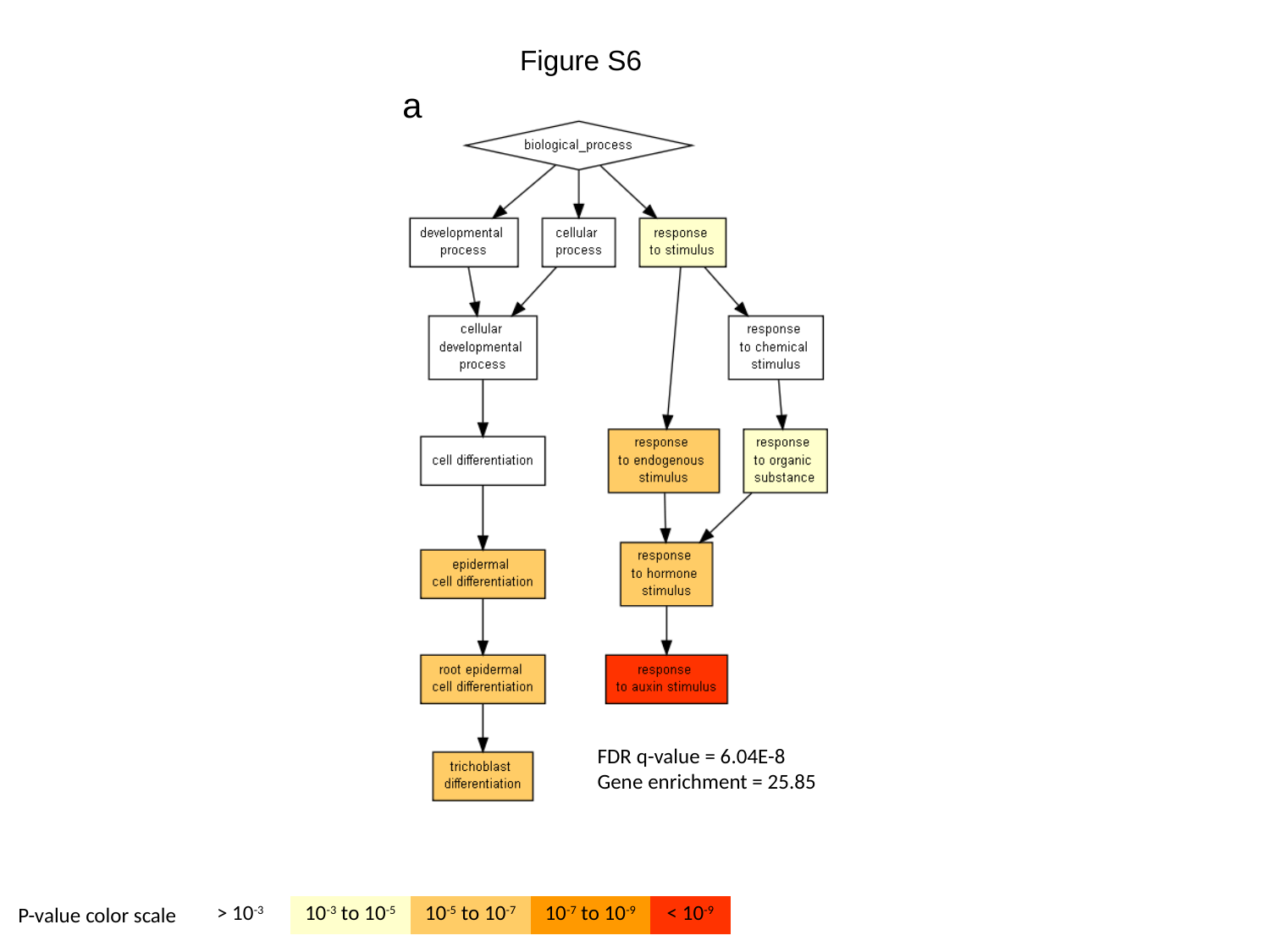

Figure S6
a
FDR q-value = 6.04E-8
Gene enrichment = 25.85
P-value color scale
| > 10-3 | 10-3 to 10-5 | 10-5 to 10-7 | 10-7 to 10-9 | < 10-9 |
| --- | --- | --- | --- | --- |

## Slide 2
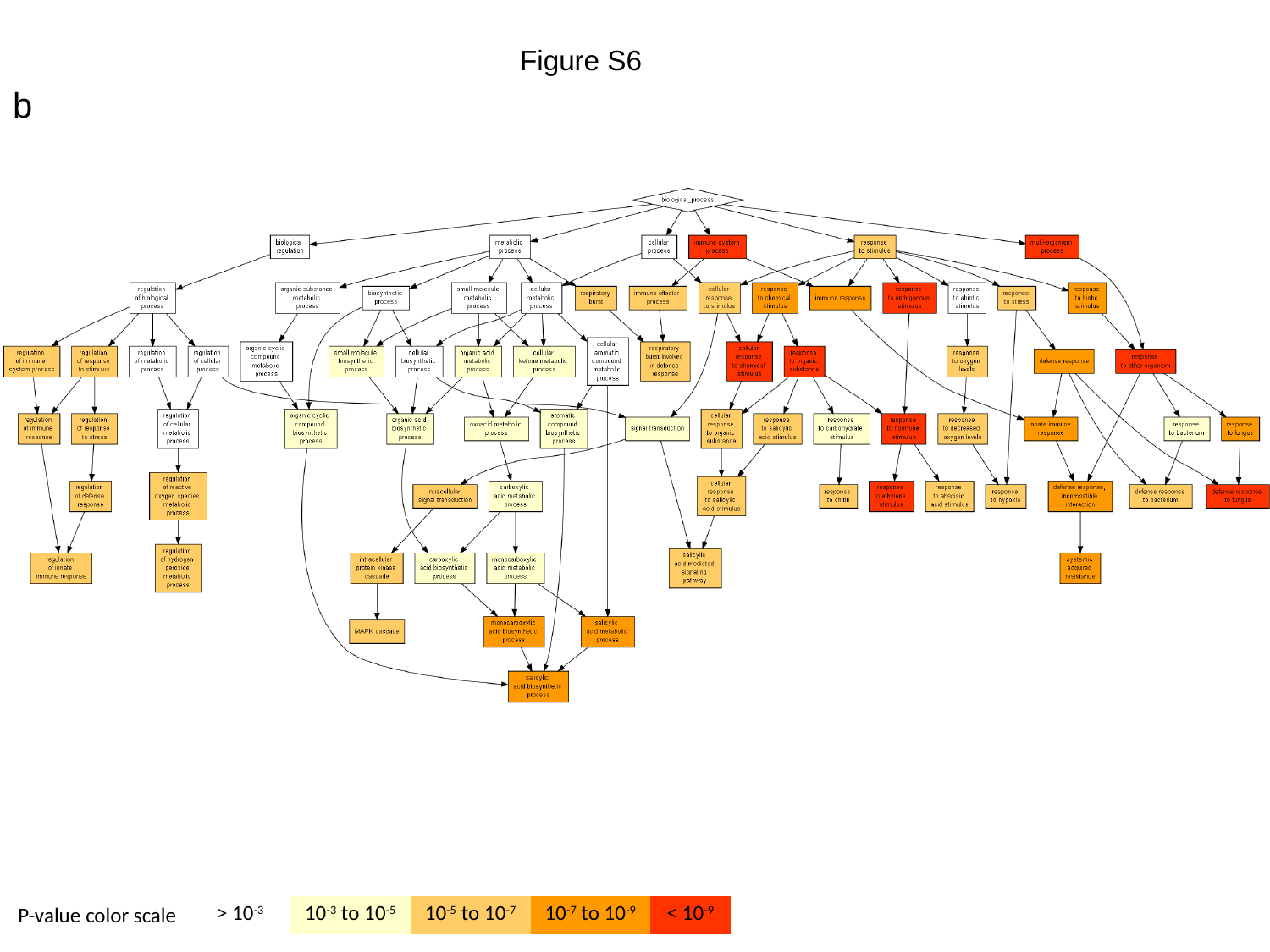

Figure S6
b
P-value color scale
| > 10-3 | 10-3 to 10-5 | 10-5 to 10-7 | 10-7 to 10-9 | < 10-9 |
| --- | --- | --- | --- | --- |
