# Supplementary material for: PORPHOBILINOGEN DEAMINASE Deficiency Alters Vegetative and Reproductive Development and Causes Lesions in Arabidopsis
Source: PLoS One. 2013 Jan 8;8(1):e53378. doi: 10.1371/journal.pone.0053378 (PMC3540089; doi:10.1371/journal.pone.0053378)
Supplement: Table S2 — Primers used in this work. (PDF) [file pone.0053378.s008.pdf]

Table S2.- Primers used in this work

Purpose	Primer names (Forward/Reverse)	Oligonucleotide sequences (5´→3´)	
		Forward primer	Reverse primer
Cycle sequencing	At5g08280-F1/R1	CAACTCCATTTGAAGCGAAATTC	GCAGGATATTTGTGGAGAATC
	At5g08280-F2/R2	AAGATGTCCCAACTTACTTACC	CGGGACTTCAAGATGTTGAATC
qRT-PCR	qAt1g74710-F/R	CGTTCGGTTACAGGTTCCAATT	GTTCTCGTTAGCTAGATCGAGA
	qAt1g75830-F/R	TGCTGCTCTTGAAGCACCGAT	TGTCGTGCTTTCTCAAGGTTAAT
	qAt2g29350-F/R	TCAACATCCTCGTCAACAATGT	GCAAGCTGTGAGAGATGGAAA
	qAt2g14610-F/R	CTCAAGATAGCCCACAAGATTAT	CTGCAGTTGCCTCTTAGTTGTT
	qAt5g10140-F/R	TTGAACTTGTGGATAGCAAGCTT	CGGTCTTCTTGGCTCTAGTCA
	qAt1g65480-F/R	GAACAACCTTTGGCAATGAGATT	CACCCTGGTGCATACACTGTT
	qAt2g45660-F/R	GCCAGCTCCAATATGCAAGATA	CTTCATATTTCAAATGCTGCATATT
	qAt3g04120-F/R	ATCACTACTGAGTACATGACCTA	TCATCCTTGATCTTGAGTTCAT
rug1 phenotypic rescue and *RUG1* overexpression	attbRUG1-OE-F/R	GGGGACAAGTTTGTACAAAAAAGCAG GCTATGGATATTGCTTCGTCATCTC	GGGGACCACTTTGTACAAGAAAGCTGGGTT CAGTTGCCGAAGAAGCCAG
